# Ocular Surface Symptoms, Objective Redness, and Intraocular Pressure After a Switch from Fixed Dorzolamide/Timolol to Netarsudil in Latanoprost-Treated Glaucoma and Ocular Hypertension: A Prospective Real-World Cohort Study

**DOI:** 10.3390/medicina62091768

**Published:** 2026-09-15

**Authors:** Virginia Zanutigh, Celina Logioco, Leila Galetto, Franco Perrone, Noelia Podesta, Florencia Valvecchia

**Affiliations:** 1 Glaucoma Service, Centro de Ojos Quilmes, Quilmes B1878KDF, Buenos Aires, Argentina; celinalogioco@gmail.com (C.L.); leilagaletto@gmail.com (L.G.); noelpodesta@gmail.com (N.P.); 2 Cornea Service, Centro de Ojos Quilmes, Gran Buenos B1878KDF, Buenos Aires, Argentina; francoaugustoperrone@gmail.com; 3 Ocular Surface Service, Centro de Ojos Quilmes, Quilmes B1878KDF, Buenos Aires, Argentina; florvalvecchia15@gmail.com

**Keywords:** netarsudil, Rhopressa, glaucoma, ocular surface disease, Ocular Surface Disease Index, conjunctival hyperemia, Keratograph 5M, intraocular pressure, treatment burden, patient preference

## Abstract

*Background and Objectives*: Conjunctival hyperemia is common with netarsudil, but redness may not reflect the overall patient experience when treatment burden is reduced. We evaluated ocular surface symptoms, objective redness, tear-film parameters, intraocular pressure (IOP), and treatment preference after a routine-care switch from twice-daily fixed dorzolamide/timolol to once-daily netarsudil 0.02%, while continuing latanoprost. *Materials and Methods*: This prospective, single-center observational cohort included 30 consecutive adults with primary open-angle glaucoma or ocular hypertension and controlled IOP. Outcomes were assessed at baseline and months 1 and 2. The primary outcome was the Ocular Surface Disease Index (OSDI). Continuous eye-level outcomes were analyzed using mixed-effects models accounting for fellow-eye and repeated-measures correlation; grade ≥ 2 hyperemia was analyzed using participant-clustered logistic regression. *Results*: Twenty-seven participants (54 eyes) completed evaluable 2-month follow-up. Median OSDI decreased from 7.50 at baseline to 6.25 at month 1 and 5.00 at month 2 (Friedman *p* = 0.0015). The mean baseline-to-month 2 change was −2.95 points (95% CI, −4.80 to −1.10), and 6/27 participants (22.2%) improved by at least 7 points. Serle grade ≥ 2 hyperemia increased from 7.4% of eyes at baseline to 51.9% at month 1 and 44.4% at month 2. Estimated mean bulbar redness index increased from 1.57 to 2.14 (*p* < 0.0001), tear meniscus height increased modestly, and non-invasive keratograph break-up time showed no significant longitudinal change. Estimated mean IOP changed from 14.50 to 13.65 mmHg (*p* = 0.0083), with no eye showing an increase of at least 4 mmHg. Burning improved after multiplicity adjustment, and 17/27 participants with available month-2 preference data (63.0%) preferred netarsudil. *Conclusions*: The switch was associated with modest OSDI improvement and maintained short-term IOP control despite increased objective redness. Patient preference appeared to reflect factors beyond redness alone.

## 1. Introduction

The appearance of the eyes matters in everyday social interaction. Unlike many treatment-related adverse effects, ocular redness is immediately visible to other people. Experimental studies of facial perception have shown that a redder scleral appearance can make a face seem less healthy, less attractive, and older [1,2]. In ordinary interactions, red eyes may therefore prompt questions or assumptions about fatigue, crying, allergy, infection, or even substance use. Such reactions may occur regardless of the actual cause of the redness. For a patient receiving chronic ophthalmic therapy, conjunctival hyperemia is thus more than a biomicroscopic finding: its visibility may influence self-consciousness, treatment acceptability, and the overall experience of care.

This concern is particularly relevant in glaucoma and ocular hypertension, for which topical intraocular pressure (IOP)-lowering therapy remains central to long-term management. Because treatment is usually lifelong and frequently involves more than one active agent, its success depends not only on pharmacologic efficacy but also on ocular tolerability, treatment burden, persistence, and correct administration. Ocular surface disease (OSD) is common among patients receiving topical glaucoma therapy, and both symptoms and objective abnormalities become more frequent or severe as the number of medications and daily instillations increases [3,4,5,6,7,8,9]. In a multicenter study of 630 medically treated patients, 48.4% reported at least mild OSD symptoms, and mean Ocular Surface Disease Index (OSDI) scores increased with the number of topical IOP-lowering medications [4].

The consequences of OSD extend beyond ocular discomfort. Burning, foreign-body sensation, fluctuating vision, and hyperemia may adversely affect quality of life, while complex schedules, difficulty instilling drops, and local adverse effects can contribute to poor adherence and treatment discontinuation [10,11]. In this study, treatment burden refers specifically to the number of active agents, the number of daily instillations, and schedule complexity; it was not assessed using a separate validated treatment-burden instrument. Lower adherence has, in turn, been associated with visual-field progression [12]. The OSDI is a validated 12-item patient-reported outcome measure that captures ocular symptoms, vision-related function, and environmental triggers on a 0–100 scale [13]. A change of approximately 7–10 points has been proposed as a minimal clinically important difference, depending on baseline severity [14]. Patient-reported outcomes are particularly relevant because the visible appearance of the ocular surface and the symptoms experienced by the patient represent related but not necessarily equivalent dimensions of treatment tolerability.

Netarsudil 0.02% is a once-daily Rho kinase inhibitor that is believed to lower IOP by increasing aqueous humor outflow through the trabecular meshwork [15,16,17]. In the ROCKET phase 3 program, once-daily netarsudil provided IOP lowering that was noninferior to twice-daily timolol in patients with baseline IOP below 25 mmHg [18,19,20]. Twenty-four-hour efficacy has also been demonstrated [21], and the complementary mechanisms of netarsudil and latanoprost have produced additive IOP lowering in the MERCURY trials [22,23].

Nevertheless, conjunctival hyperemia is the most frequently reported netarsudil-associated adverse event, occurring in approximately one-half of participants in pivotal trials [18,19,20]. This creates a clinically relevant trade-off. When netarsudil replaces part of a multidrug regimen, patients may experience greater visible redness while simultaneously benefiting from fewer active agents and fewer daily instillations. Clinical trials have carefully characterized the incidence and severity of hyperemia, but they provide comparatively limited information about how patients perceive this trade-off in routine care. In particular, it remains unclear whether an objectively redder ocular surface necessarily translates into worse OSD symptoms, lower treatment acceptability, or a preference for the previous, more complex regimen.

Accordingly, the present study examined not only whether the eyes became redder after switching to netarsudil, but also how patients experienced the change. The primary objective was to evaluate the change in OSDI after switching patients with controlled IOP from latanoprost once daily plus fixed-dose dorzolamide/timolol twice daily to latanoprost once daily plus netarsudil 0.02% once daily. Secondary objectives were to evaluate slit-lamp and instrument-based conjunctival redness, tear-film parameters, IOP, treatment persistence, comparative ocular symptoms, and patient preference over 2 months.

## 2. Materials and Methods

### 2.1. Study Design and Setting

This was a prospective, single-center, observational cohort study with within-patient longitudinal comparisons conducted at Centro de Ojos Quilmes, Quilmes, Buenos Aires, Argentina. Following regulatory approval and local availability, netarsudil was incorporated into routine glaucoma care as a therapeutic option for clinically suitable patients with controlled IOP. In these patients, treating glaucoma specialists considered replacing fixed dorzolamide 2%/timolol 0.5% twice daily with netarsudil 0.02% once daily to reduce the number of active agents and daily instillations while maintaining latanoprost 0.005% once daily.

For each patient, the treatment decision was made by the treating ophthalmologist on clinical grounds and was not contingent on participation in the study. The research component consisted of the prospective and standardized collection and analysis of outcomes following this routine-care treatment change. The cohort comprised the first 30 consecutive eligible patients who underwent the switch between 1 December 2025 and 31 January 2026. The 2-month follow-up of the last included patient was completed in March 2026.

Clinical outcomes were assessed immediately before the treatment change and approximately 1 and 2 months thereafter. Ocular surface questionnaires, slit-lamp examination, IOP measurement, and Keratograph 5M assessments formed part of the standardized clinical evaluation used by the glaucoma service. Beyond the clinically selected substitution of fixed dorzolamide/timolol with netarsudil, the research protocol did not mandate the initiation, withdrawal, or modification of any ocular treatment. All subsequent treatment decisions remained under the responsibility of the treating ophthalmologist and followed the usual clinical practice of the glaucoma service.

Database validation and statistical analysis were performed by the Data Analysis and Statistics Service of Centro de Ojos Quilmes, independently of the clinical care team. Analysts had not participated in patient inclusion, treatment decisions, clinical examinations, or outcome collection. During database validation and finalization of the statistical analysis specifications, visits were labeled with nonchronological codes so that the analysts were unaware of whether a record corresponded to baseline, month 1, or month 2. The code-to-visit mapping was disclosed only after the database and analysis specifications had been finalized.

### 2.2. Ethics and Reporting Standards

The study was conducted in accordance with the Declaration of Helsinki and was approved by the institutional ethics committee of Centro de Ojos Quilmes (approval number RhoArgCOC090925; approval date: 9 September 2025). All participants provided written informed consent for the prospective collection and scientific analysis of their clinical data. Because treatment decisions were made as part of routine clinical care and were not assigned for research purposes, this observational cohort was not registered as a clinical trial. The manuscript was prepared in accordance with the Strengthening the Reporting of Observational Studies in Epidemiology (STROBE) statement [24].

### 2.3. Participants

Adults aged 18 years or older were eligible if they had primary open-angle glaucoma or ocular hypertension, with a stable, controlled IOP of 21 mmHg or lower while receiving latanoprost 0.005% once daily plus fixed dorzolamide 2%/timolol 0.5% twice daily. Patients also had to be considered clinically suitable by their treating ophthalmologist for replacement of fixed dorzolamide/timolol with netarsudil 0.02% once daily.

No minimum or maximum baseline OSDI score was required because the study was intended to evaluate ocular-surface outcomes across the full spectrum of patient-reported symptom severity. Patients with any baseline OSDI score could therefore be included, provided that they had not used artificial tears, ocular lubricants, topical vasoconstrictors or redness-relief drops, or any other topical treatment for dry eye during the preceding 3 months. This treatment-free period predated study inclusion and did not represent a protocol-mandated washout.

Other exclusion criteria were active uveitis; contact-lens wear; previous herpetic keratitis or another chronic keratopathy; conjunctivitis within the preceding 3 months; glaucoma surgery or laser treatment within the preceding 3 months; other ocular surgery within the preceding 6 months; chronic use of any other topical ophthalmic medication; pregnancy or breastfeeding; and known hypersensitivity to netarsudil.

For descriptive purposes, glaucoma stage was extracted from the clinical record and categorized as mild, moderate, or severe according to the routine clinical classification used by the glaucoma service. Ocular hypertension was recorded as a separate diagnostic category. Disease stage was not used as an eligibility criterion or for subgroup analysis.

### 2.4. Clinical Treatment Change and Follow-Up

As part of the routine-care treatment change previously selected by the treating ophthalmologist, fixed dorzolamide 2%/timolol 0.5% twice daily was discontinued after completion of the baseline assessment, and netarsudil 0.02% once daily in the evening (Rhopressa^®^; Alcon, Fort Worth, TX, USA) was initiated. Latanoprost 0.005% once daily was continued without modification.

The regimen therefore changed from three active agents administered in three daily instillations to two active agents administered in two daily instillations. A standardized remote safety contact was conducted on day 14 (±3 days) to assess early tolerability, including conjunctival hyperemia, ocular pain, blurred vision, adherence, and treatment continuation. If severe symptoms, a desire to discontinue netarsudil, or another safety concern was identified, an in-person examination was arranged within 24 h.

Patients were advised to contact the center before making any treatment change whenever possible. Predefined clinical safety findings prompting reassessment and consideration of netarsudil discontinuation included an IOP increase greater than 4 mmHg from baseline; grade 3 conjunctival hyperemia accompanied by pain or photophobia; loss of more than two lines of best-corrected distance visual acuity; or any other clinically important event according to the treating ophthalmologist. The final decision to discontinue netarsudil or resume the previous regimen remained clinical and individualized.

During follow-up, participants were not instructed to avoid ocular-surface treatment. Artificial tears, ocular lubricants, or another dry-eye treatment could be initiated at any time if requested by the patient or considered clinically indicated by the treating ophthalmologist. Any such treatment, including its indication and timing, was to be recorded and its potential influence on ocular-surface outcomes considered individually. Use of topical vasoconstrictors or over-the-counter redness-relief drops was also to be documented. Participants were informed that conjunctival redness was an expected effect of netarsudil and were instructed to report pain, photophobia, visual deterioration, intolerable symptoms, or any other concern. Counseling about expected redness did not replace clinical reassessment or treatment when indicated.

### 2.5. Outcomes and Assessments

#### 2.5.1. Primary Outcome: OSDI

Participants completed a Spanish-language version of the validated 12-item OSDI questionnaire at baseline, month 1, and month 2 [13]. Total scores range from 0 to 100, with higher scores indicating greater ocular surface symptom burden. Scores were categorized as normal (≤12), mild (>12 to 22), moderate (>22 to 32), or severe (>32), using established OSDI severity thresholds [14]. The primary outcome was change in total OSDI score during follow-up. A secondary responder analysis estimated the proportion of participants with a reduction of at least 7 points between baseline and month 2, using this threshold as a clinically meaningful improvement [14].

#### 2.5.2. Slit-Lamp Examination

Conjunctival hyperemia was graded separately in each eye at every visit using the four-point scale employed in the netarsudil clinical development program: 0, none or normal; 1, mild; 2, moderate; and 3, severe [18]. Hyperemia grades were retained as eye-level observations for analysis.

Slit-lamp examination also assessed eyelid erythema or edema, corneal edema, corneal staining, anterior chamber cells or flare, and lens opacity in phakic eyes. Slit-lamp examination also assessed eyelid erythema or edema, conjunctival hemorrhage, corneal verticillata, corneal edema, corneal staining, anterior chamber cells or flare, and lens opacity in phakic eyes. Best-corrected distance visual acuity was recorded in Snellen units and converted to logarithm of the minimum angle of resolution (logMAR) for analysis.

#### 2.5.3. Keratograph 5M Measurements

Objective ocular surface measurements were obtained using the Keratograph 5M (OCULUS Optikgeräte GmbH, Wetzlar, Germany). Measurements were recorded separately in both eyes whenever the eyes were eligible and technically assessable. All eligible eyes with available longitudinal data were included in the eye-level analyses; no single study eye was selected post hoc.

Bulbar redness was assessed nasally and temporally using the automated R-Scan module. Nasal and temporal measurements were averaged within each eye to obtain the bulbar redness index (BRI). The same device was used to measure non-invasive Keratograph tear-film break-up time (NIKBUT) and central lower tear meniscus height (TMH).

The NIKBUT average value was selected for analysis. This parameter represents the average tear-film break-up time across the corneal regions analyzed during the acquisition period, rather than the time to the first detected tear-film disruption. Both NIKBUT-first and NIKBUT-average have demonstrated acceptable repeatability and reproducibility with the Keratograph 5M [25].

Keratograph assessments were performed before applanation tonometry, fluorescein instillation, or other contact ocular procedures and under standardized examination conditions. The Keratograph 5M automated redness module has previously been used to quantify medication-associated ocular redness in patients receiving topical glaucoma therapy [26].

#### 2.5.4. Intraocular Pressure Measurement

IOP was measured separately in each eye between 10:00 and 12:00 at every in-person visit using calibrated Goldmann applanation tonometry. Three consecutive measurements were obtained in each eye, and their mean was used for analysis. For each participant, follow-up measurements were scheduled within the same time window as the baseline measurement whenever possible to minimize the influence of diurnal variation.

#### 2.5.5. Persistence, Comparative Symptoms, and Treatment Preference

Persistence was defined as continued netarsudil use through the 2-month visit. Dates and reasons for discontinuation were recorded. At month 2, participants rated itching, foreign-body sensation, burning, blurred vision, and red eye for both netarsudil and their previous fixed dorzolamide/timolol drop on a scale from 0 to 5, with higher scores indicating greater severity. When bilateral numerical responses were available, the two values were averaged within the participant; when only one eye-specific response was available, that value was retained. Symptoms were classified as improved, unchanged, or worsened according to the direction of the participant-level score difference. Participants also identified which drop had caused greater overall ocular discomfort and stated whether they preferred fixed dorzolamide/timolol, netarsudil, or had no preference.

### 2.6. Sample Size

Sample-size planning was based on the clinically relevant within-participant change in OSDI between baseline and month 2. Assuming a 7-point mean paired difference, a standard deviation of the paired change of 10 points, a two-sided alpha level of 0.05, and 80% power, a minimum of 19 participants with complete paired observations was required. Because the study was embedded in the initial clinical implementation of netarsudil at the study center, a target of 30 consecutive eligible patients was prespecified before enrollment to improve estimation precision beyond the calculated minimum while maintaining feasible and standardized follow-up. Recruitment stopped when this target was reached, and consecutive inclusion was used to reduce investigator selection within the implementation cohort. No formal inflation for attrition was applied. The study was not powered for subgroup analyses or for definitive inference regarding secondary and exploratory outcomes.

### 2.7. Statistical Analysis

Categorical variables were summarized as counts and percentages. Continuous variables were summarized as mean ± standard deviation when approximately normally distributed and as median with interquartile range (IQR) when distributions were skewed.

Participant-level OSDI scores were compared across baseline, month 1, and month 2 using the Friedman test, restricted to participants with complete repeated measurements. Kendall’s W was reported as an effect-size measure. Pairwise comparisons were performed using Wilcoxon signed-rank tests, with Holm adjustment across the three visit contrasts. Mean within-participant changes were reported with 95% confidence intervals (CIs). The proportion achieving an OSDI reduction of at least 7 points between baseline and month 2 was reported with a Wilson 95% CI.

To account simultaneously for correlation between fellow eyes within the same participant and repeated measurements within each eye, Keratograph measurements, IOP, and logMAR visual acuity were analyzed as eye-level longitudinal outcomes using linear mixed-effects models. Visit was included as a categorical fixed effect, with random intercepts for participants and for eyes nested within participants. Model-based contrasts were used to compare visits. Fellow eyes were therefore not treated as statistically independent observations [27]. Grade ≥ 2 hyperemia was analyzed using a marginal logistic model with participant-clustered robust standard errors.

OSDI severity categories, the full slit-lamp hyperemia distribution, treatment persistence, adverse events, comparative symptoms, and treatment preference were summarized descriptively. Paired symptom scores were compared using Wilcoxon signed-rank tests, with Holm adjustment across the five symptom comparisons. For exploratory symptom-sign analyses, bilateral objective measurements were averaged within each participant, and Spearman correlations were calculated between OSDI and ocular-surface measurements at each visit and between their changes from baseline to month 2; Holm adjustment was applied across the correlation set.

Except for the prespecified families of OSDI pairwise contrasts, symptom comparisons, and exploratory correlations described above, no multiplicity adjustment was applied to secondary analyses; these *p*-values were interpreted as exploratory. Mixed-effects models incorporated all available eye-level measurements, whereas Friedman analyses were restricted to participants with complete repeated OSDI observations. Statistical significance was defined as a two-sided *p* < 0.05. Analyses were performed using IBM SPSS Statistics version 27 (IBM Corp., Armonk, NY, USA).

## 3. Results

### 3.1. Participant Flow and Data Completeness

Thirty consecutive participants, contributing 60 eyes, underwent the treatment switch from fixed dorzolamide/timolol to netarsudil while continuing latanoprost. Three participants did not complete an evaluable 2-month follow-up: one was lost to follow-up after baseline, one did not return after the 1-month visit because of work-related circumstances, and one experienced a family emergency and did not maintain the prescribed treatment regimen. Therefore, 27/30 participants (90.0%), contributing 54 eyes, completed the 2-month follow-up and were included in the longitudinal comparative analyses. Mean participant age was 47.4 ± 9.2 years, and 17/30 participants (56.7%) were women. At baseline, 10/60 eyes (16.7%) had mild glaucoma, 25/60 (41.7%) moderate glaucoma, 22/60 (36.7%) severe glaucoma, and 3/60 (5.0%) ocular hypertension. Among the 27 completers, OSDI observations were available at all three visits, and Serle hyperemia grades, IOP, and BCVA were available for all 162 potentially available eye visits. Following data validation, 159 BRI observations and 160 NIKBUT and TMH observations were retained for the mixed-model analyses. Missing values were not imputed.

### 3.2. Ocular Surface Disease Index

**Figure 1:**
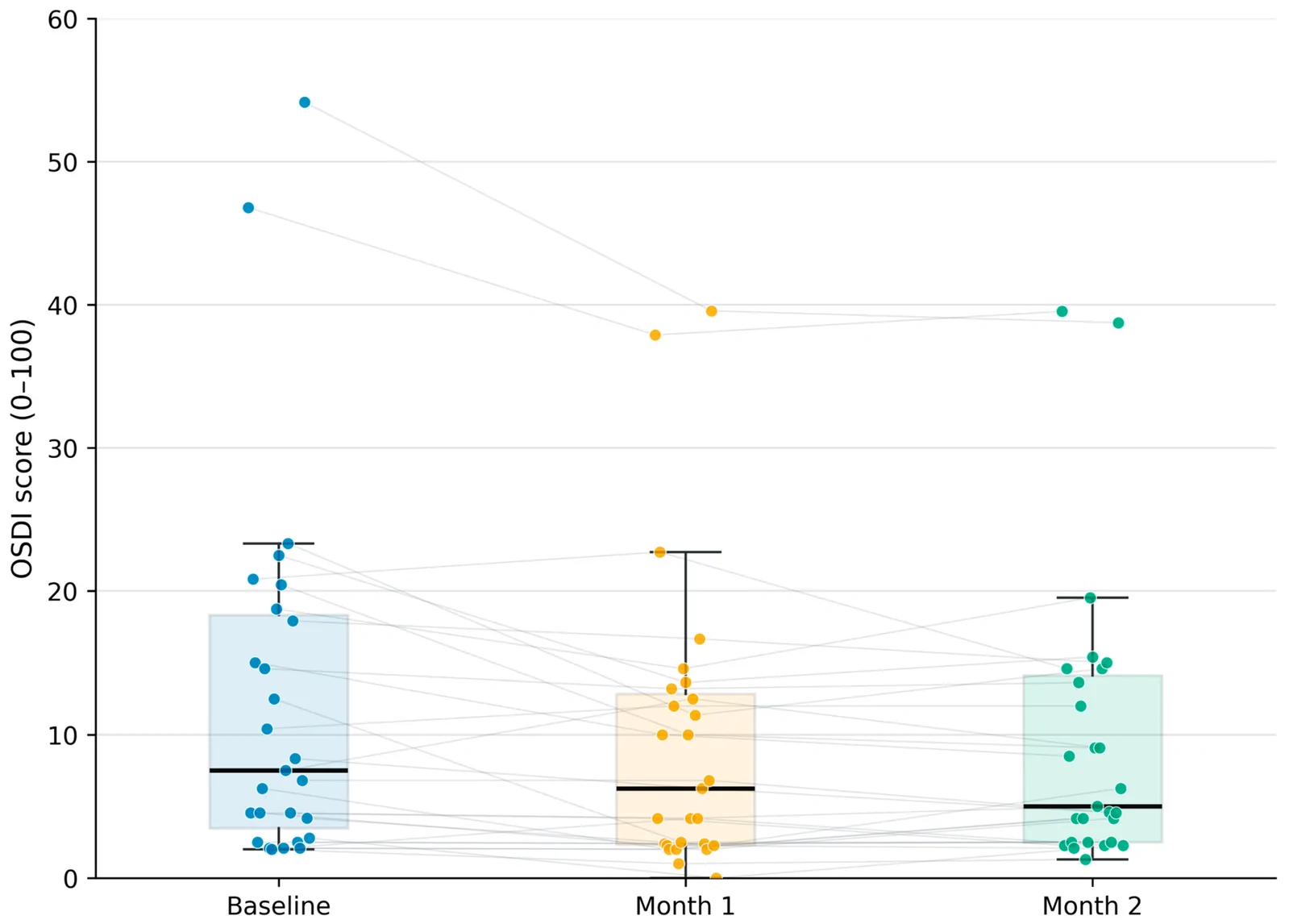
Participant-level Ocular Surface Disease Index (OSDI) scores at baseline and months 1 and 2 after the switch to netarsudil (*n* = 27). Points and connecting lines show individual trajectories; boxes show the interquartile range and median, and whiskers extend to 1.5 times the interquartile range.

OSDI scores decreased during follow-up (Figure 1). Median OSDI was 7.50 (IQR, 3.48–18.33) at baseline, 6.25 (IQR, 2.34–12.85) at month 1, and 5.00 (IQR, 2.50–14.11) at month 2. The corresponding mean ± SD values were 12.59 ± 13.08, 9.57 ± 10.19, and 9.64 ± 9.98, respectively.

The overall longitudinal difference was statistically significant (Friedman χ^2^(2) = 13.07; *p* = 0.0015; Kendall’s W = 0.242). Mean change from baseline was −3.01 points at month 1 (95% CI, −4.91 to −1.12; Wilcoxon *p* = 0.0022; Holm-adjusted *p* = 0.0066) and −2.95 points at month 2 (95% CI, −4.80 to −1.10; Wilcoxon *p* = 0.0035; Holm-adjusted *p* = 0.0070). There was no significant additional change between months 1 and 2 (Holm-adjusted *p* = 0.619).

The proportion of participants in the normal OSDI category increased from 16/27 (59.3%) at baseline to 19/27 (70.4%) at both month 1 and month 2. Severe OSDI was present in 2/27 participants (7.4%) at each visit. The two participants classified as having severe OSDI at baseline had not used ocular lubricants, topical vasoconstrictors, or any other dry-eye treatment during the preceding 3 months. Their OSDI trajectories were 46.80, 37.90, and 39.54 and 54.16, 39.58, and 38.75 at baseline, month 1, and month 2, respectively. Both therefore achieved a reduction of at least 7 points by month 2, although their scores remained within the severe category. Six participants (22.2%; 95% CI, 10.6–40.8%) achieved a clinically important OSDI reduction of at least 7 points by month 2. No participant experienced an increase of at least 7 points.

### 3.3. Conjunctival Hyperemia and Tear-Film Measurements

**Figure 2:**
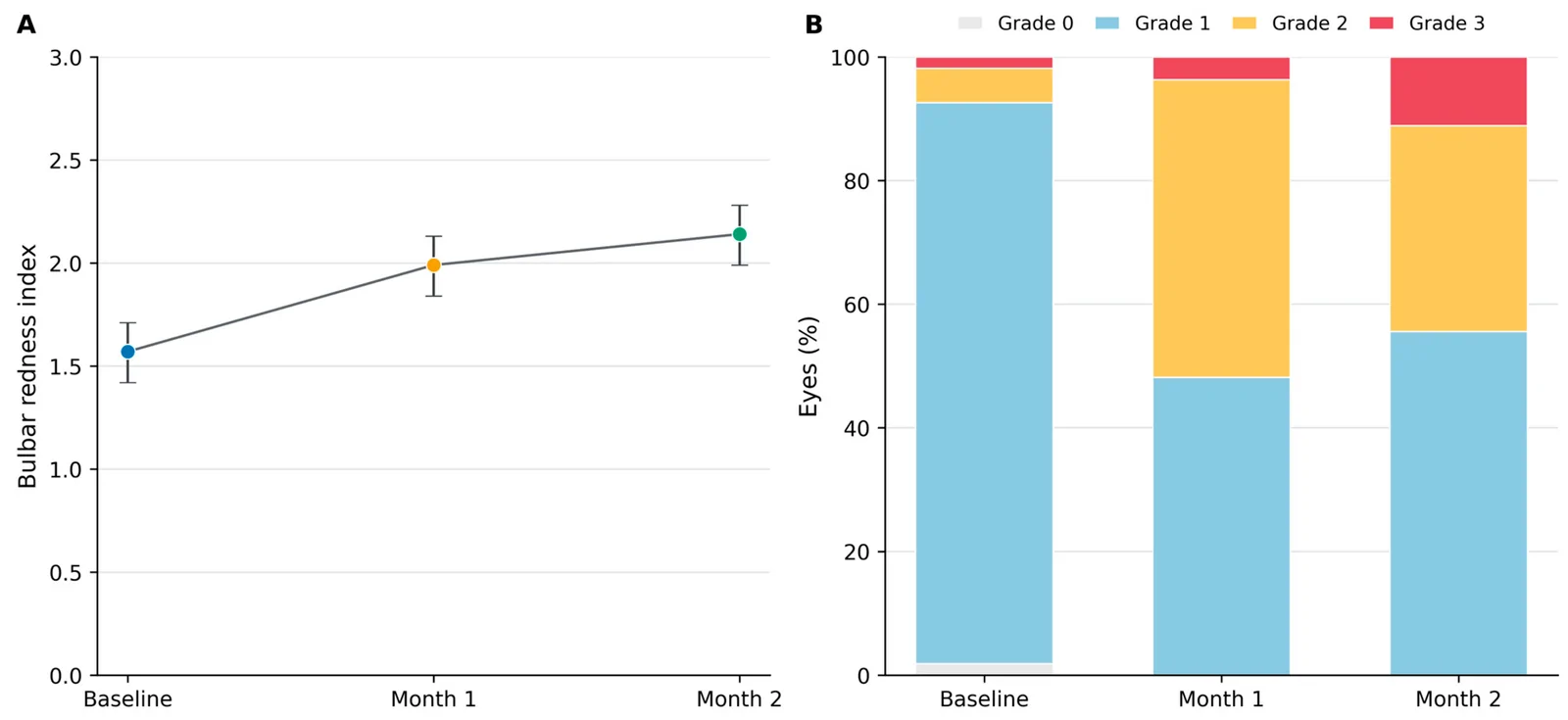
**Conjunctival hyperemia following the switch to netarsudil.** (**A**) Linear mixed-model estimated mean bulbar redness index (BRI) with 95% confidence intervals at baseline and months 1 and 2. (**B**) Distribution of Serle conjunctival hyperemia grades among 54 eyes at each visit.

Slit-lamp and instrument-based assessments both demonstrated increased conjunctival redness after the treatment switch (Table 1 and Figure 2). Serle grade 2 or 3 hyperemia was present in 4/54 eyes (7.4%) at baseline, 28/54 (51.9%) at month 1, and 24/54 (44.4%) at month 2. Grade 3 hyperemia was observed in 1/54 eyes (1.9%) at baseline, 2/54 (3.7%) at month 1, and 6/54 (11.1%) at month 2.

In the participant-clustered logistic model, the odds of grade ≥ 2 hyperemia were higher at month 1 than at baseline (OR, 13.46; 95% CI, 5.04–35.92; *p* < 0.0001) and remained higher at month 2 (OR, 10.00; 95% CI, 3.18–31.40; *p* < 0.0001). There was no significant difference between months 1 and 2 (OR, 0.74; 95% CI, 0.40–1.39; *p* = 0.3513).

Mixed-model estimated mean BRI increased from 1.57 at baseline to 1.99 at month 1 and 2.14 at month 2 (overall *p* < 0.0001). The estimated increase was 0.42 at month 1 (95% CI, 0.29–0.54; *p* < 0.0001) and 0.57 at month 2 (95% CI, 0.44–0.70; *p* < 0.0001). BRI was also slightly higher at month 2 than at month 1 (difference, 0.15; 95% CI, 0.03–0.28; *p* = 0.0172).

TMH increased from an estimated mean of 0.275 mm at baseline to 0.359 mm at month 1 and 0.329 mm at month 2 (overall *p* < 0.0001). Compared with baseline, the estimated increase was 0.084 mm at month 1 (95% CI, 0.046–0.121; *p* < 0.0001) and 0.054 mm at month 2 (95% CI, 0.017–0.091; *p* = 0.0041). By contrast, NIKBUT did not demonstrate a sustained longitudinal change. Estimated means were 7.17 s at baseline, 8.76 s at month 1, and 7.34 s at month 2 (overall *p* = 0.2154).

### 3.4. Intraocular Pressure and Visual Acuity

Mean IOP remained controlled throughout follow-up. Mixed-model estimated means were 14.50 mmHg at baseline, 14.33 mmHg at month 1, and 13.65 mmHg at month 2 (overall visit effect, *p* = 0.0083). There was no significant difference between baseline and month 1 (estimated difference, −0.17 mmHg; 95% CI, −0.74 to 0.40; *p* = 0.568). At month 2, the mean IOP was slightly lower than at baseline (estimated difference, −0.85 mmHg; 95% CI, −1.42 to −0.28; *p* = 0.0035). No eye experienced an IOP increase of 4 mmHg or greater from baseline at either follow-up visit.

Mixed-model estimated mean BCVA changed from 0.142 logMAR at baseline to 0.127 at month 1 and 0.124 at month 2 (overall visit effect, *p* = 0.0215). Although this small numerical improvement reached statistical significance, its magnitude was not considered clinically relevant. No eye experienced a deterioration of 0.2 logMAR or greater during follow-up. No corneal edema, clinically relevant corneal staining, anterior chamber inflammation, eyelid edema or erythema, or other serious ocular adverse event was identified.

No participant used artificial tears, ocular lubricants, another topical dry-eye treatment, topical vasoconstrictors, or over-the-counter redness-relief drops during follow-up. Participants with hyperemia received counseling and clinical monitoring, and none initiated additional ocular-surface therapy during the 2-month observation period.

### 3.5. Safety, Treatment Persistence, and Concomitant Ocular-Surface Therapy

All 27 participants with evaluable month-2 follow-up remained on netarsudil through that visit. No treatment discontinuation attributed to conjunctival hyperemia or another adverse event was documented. The three noncompleters did not provide month-2 safety or preference data; their reasons for noncompletion are reported in Section 3.1. No corneal edema, clinically relevant corneal staining, anterior chamber inflammation, eyelid edema or erythema, or serious or unexpected ocular adverse event was identified.

Among the 27 completers, no participant initiated or used artificial tears, ocular lubricants, another topical dry-eye treatment, topical vasoconstrictors, or over-the-counter redness-relief drops during follow-up. Because no concomitant ocular-surface therapy was introduced, no sensitivity analysis excluding or adjusting for such treatment was required. No corneal verticillata, conjunctival hemorrhage, or grade 3 hyperemia accompanied by pain or photophobia was documented, and no day-14 safety contact triggered an unscheduled examination.

### 3.6. Comparative Symptoms and Treatment Preference

All 27 participants who completed the 2-month follow-up completed the final questionnaire (Table 2). Burning was more frequently rated as improved with netarsudil (12/27, 44.4%) than worsened (1/27, 3.7%). Its mean score decreased from 1.39 with the previous dorzolamide/timolol drop to 0.39 with netarsudil (Wilcoxon *p* = 0.0049; Holm-adjusted *p* = 0.024). Itching also showed a nominal reduction (mean score, 1.33 versus 0.80; *p* = 0.018), although this comparison did not remain statistically significant after adjustment for multiple comparisons (adjusted *p* = 0.073).

Foreign-body sensation and blurred vision did not differ significantly between drops. Red eye was more frequently rated as worsened (10/27, 37.0%) than improved (2/27, 7.4%). The mean red-eye score increased from 0.46 with the previous drop to 0.89 with netarsudil, although the paired difference was not statistically significant (*p* = 0.096; adjusted *p* = 0.287).

When asked which drop had caused more overall ocular discomfort, 10/27 participants (37.0%) selected the previous dorzolamide/timolol drop, 2/27 (7.4%) selected netarsudil, and 15/27 (55.6%) reported no overall difference. Among the 27 participants with available preference data, 17 (63.0%) preferred netarsudil, 9 (33.3%) had no preference, and 1 (3.7%) preferred the previous drop. Thus, 17/30 participants in the fully enrolled cohort (56.7%) were documented as preferring netarsudil, whereas preference remained unknown for the three noncompleters.

### 3.7. Associations Between Symptoms and Objective Findings

None of the exploratory correlations were statistically significant before or after adjustment for multiple comparisons. At month 2, OSDI was not significantly correlated with mean BRI (Spearman ρ = −0.205; unadjusted *p* = 0.305) or mean Serle grade (ρ = 0.051; unadjusted *p* = 0.802). The correlation between change in OSDI and change in mean BRI from baseline to month 2 was positive but did not reach statistical significance (ρ = 0.345; unadjusted *p* = 0.078). These exploratory analyses did not identify a consistent association between objectively measured conjunctival redness and patient-reported ocular-surface symptoms.

## 4. Discussion

This prospective real-world cohort evaluated the patient experience and ocular outcomes after a routine-care switch from twice-daily fixed dorzolamide/timolol to once-daily netarsudil while latanoprost was continued. Four findings are central. First, OSDI scores decreased significantly but modestly over 2 months, with a mean improvement below the 7-point threshold used for clinical relevance. Second, slit-lamp grading and automated measurement showed a clear increase in conjunctival redness. Third, short-term IOP control was maintained, with a small reduction in estimated mean IOP at month 2 and no eye showing an increase of at least 4 mmHg. Fourth, despite greater redness, among the 27 participants with month-2 preference data, 17 preferred netarsudil, whereas preference remained unknown for the three noncompleters.

The OSDI finding requires a balanced interpretation. Median OSDI decreased from 7.50 to 5.00, and the longitudinal comparison was statistically significant, but the mean baseline-to-month 2 improvement was only 2.95 points. Six participants (22.2%) met the 7-point responder threshold, while none worsened by at least 7 points. The low baseline median also indicates a floor effect: most participants began in the normal OSDI category and therefore had limited scope for a large numerical improvement. The data consequently support the absence of overall symptomatic deterioration and a possible modest benefit, rather than a broad clinically important improvement in every patient.

The increase in hyperemia was expected from the pharmacologic and safety profile of netarsudil. Conjunctival hyperemia has been reported in approximately one-half of once-daily netarsudil recipients in pivotal trials, and longer follow-up has shown that it is commonly mild or intermittent [18,19,20]. In this cohort, grade ≥ 2 hyperemia increased from 7.4% of eyes at baseline to 51.9% at month 1 and 44.4% at month 2; grade 3 hyperemia was present in 11.1% at month 2. Automated BRI increased continuously through month 2. The concordance between the slit-lamp and instrument-based results strengthens the conclusion that the switch produced a genuine redness signal rather than an isolated grading fluctuation. Automated Keratograph assessment has likewise detected medication-associated hyperemia in other glaucoma populations [26].

An issue warranting clarification is that two participants had severe OSDI scores at baseline despite not receiving ocular-surface treatment. Neither participant had ocular-surface findings that, in the treating ophthalmologist’s judgment, required immediate topical treatment at baseline. The OSDI is a patient-reported measure of ocular symptoms, vision-related function, and environmental triggers and should not be interpreted as a stand-alone clinical diagnosis or an automatic indication for topical treatment [13]. Discordance between ocular-surface symptoms and clinical signs is also well recognized [28,29,30,31,32]. No OSDI eligibility threshold was applied because the study was intended to evaluate the full spectrum of baseline symptom burden and because the routine-care treatment switch was considered clinically acceptable regardless of baseline OSDI. Both participants with severe baseline OSDI achieved reductions of at least 7 points by month 2, although they remained within the severe category.

Importantly, the absence of ocular-surface treatment was not imposed by the study. These participants had already remained without lubricants, vasoconstrictors, or other dry-eye therapy for at least 3 months before inclusion, and treatment could be initiated at any time during follow-up if requested or considered clinically indicated. Ultimately, no participant used additional ocular-surface therapy. Patients with hyperemia were counseled that redness was an expected netarsudil-associated effect and were monitored clinically. The absence of such co-interventions reduces one potential source of confounding when interpreting the symptom and tear-film trajectories, but it should not be interpreted as advocating non-treatment of clinically significant OSD.

The regimen changed from three active agents administered in three daily instillations to two agents administered in two daily instillations. A lower number of active agents and daily instillations could plausibly contribute to improved burning or comfort [3,4,5,6,7,8,9,28,29,30,31], even as netarsudil-associated hyperemia increased. Burning improved after adjustment for multiple comparisons, whereas red eye was more often rated as worsened; OSDI decreased modestly, and neither month-2 OSDI nor change in OSDI correlated significantly with objective redness. Because OSDI integrates discomfort, vision-related function, and environmental triggers, visible redness and overall symptom burden need not change in parallel [13,32]. This interpretation remains exploratory because the study did not randomize regimen burden, directly measure adherence, or isolate the effects of individual active ingredients or formulations.

The tear-film measurements also did not indicate uniform deterioration across ocular-surface domains. TMH increased modestly, whereas NIKBUT showed no sustained change. The TMH increase may reflect reflex tearing, measurement variability, altered topical exposure, or a genuine increase in aqueous tear volume, and its clinical importance is uncertain. Prior work in glaucoma has similarly shown that symptoms, redness, TMH, NIKBUT, and staining can change in different directions [31]. These findings support multidimensional assessment rather than use of conjunctival redness as a surrogate for the entire ocular surface.

IOP findings provide short-term reassurance but should not be interpreted as evidence of equivalence or noninferiority. Estimated mean IOP decreased by 0.85 mmHg at month 2, no eye had an increase of at least 4 mmHg, and no follow-up value exceeded 21 mmHg. These results are biologically plausible given the conventional outflow mechanism of netarsudil and its demonstrated efficacy as monotherapy, in fixed combination with latanoprost, and in real-world adjunctive use [15,16,17,18,19,20,21,22,23,33,34,35,36,37]. Direct comparisons with add-on studies are limited because participants in the present cohort had already-controlled IOP and underwent substitution rather than addition of therapy.

Treatment preference offers a pragmatic complement to efficacy and safety outcomes. Among the 27 participants with month-2 data, 17 preferred netarsudil, nine had no preference, and one preferred fixed dorzolamide/timolol. The questionnaire did not ask participants to rank the reasons for their choice; therefore, a causal explanation cannot be established. Nevertheless, three concurrent findings provide a plausible interpretation: the regimen required one fewer active agent and one fewer daily instillation, 12/27 participants reported less burning, and more participants attributed greater overall discomfort to the previous drop than to netarsudil (10/27 versus 2/27). Thus, convenience and reduced burning may have outweighed cosmetic redness for some patients. Preference was unavailable for the three noncompleters and may therefore be influenced by survivorship and recall bias. None of these participants was documented as discontinuing because of hyperemia or another adverse event, although the participant lost after baseline could not be fully assessed for subsequent tolerability.

No clinically relevant loss of visual acuity, corneal edema, anterior chamber inflammation, or other serious ocular adverse event was observed. This is reassuring because netarsudil has been associated with uncommon reticular epithelial corneal edema, particularly in eyes with pre-existing corneal compromise or after ocular procedures [38,39]. The cohort and follow-up were nevertheless too small and short to estimate the frequency of uncommon adverse events.

The study has several strengths. It evaluated a clinically relevant substitution rather than de novo monotherapy, prospectively standardized follow-up, used each participant as his or her own baseline control, and combined a validated patient-reported outcome with slit-lamp grading, automated redness measurement, tear-film assessment, IOP, visual acuity, and preference. Both eyes were retained and analyzed using models that accounted for fellow-eye and repeated-measure correlation, avoiding the inappropriate assumption of independent eye-level observations [27]. The parallel assessment of patient-reported and objective outcomes revealed a clinically informative symptom-sign divergence.

Several limitations should frame interpretation. This was a single-center, nonrandomized observational cohort without a concurrent control group and with only 2 months of follow-up; causality cannot be established, and regression to the mean, expectation effects, seasonal variation, and other temporal influences cannot be excluded. Although the 27 complete OSDI series exceeded the minimum sample required under the original planning assumptions, the cohort remained modest, and the loss of three participants reduced precision and may have introduced attrition bias. The early-implementation cohort was relatively young (mean age, 47.4 years), and 47/60 eyes (78.3%) had moderate or severe glaucoma; together with the single-center setting, this case mix may limit extrapolation to older, more comorbid, or differently treated populations. Low baseline OSDI produced a floor effect. Three BRI observations and two NIKBUT and TMH observations failed data validation, although mixed models retained all valid observations; missing values were not imputed. Clinical examiners were not masked to treatment or visit. The comparative questionnaire relied partly on recall of the previous drop and was administered only to completers; preference estimates may therefore be affected by survivorship and recall bias. Multiple secondary outcomes were examined, and analyses without multiplicity correction should be considered exploratory.

Future controlled studies should compare treatment-switch strategies over longer follow-up, document formulation and preservative exposure precisely, incorporate adherence measures, and evaluate whether reduced instillation burden mediates changes in ocular-surface symptoms or treatment preference. In parallel, emerging translational approaches, including extracellular-vesicle-associated microRNAs as candidate biomarkers of corneal health and disease and extracellular-vesicle-based ocular drug-delivery platforms, may eventually broaden how ocular-surface effects are characterized and treated [40,41]. Patient-reported and objective redness outcomes should both be retained, because the present results indicate that they capture complementary rather than interchangeable dimensions of tolerability.

## 5. Conclusions

In this prospective real-world cohort, switching from twice-daily fixed dorzolamide/timolol to once-daily netarsudil while continuing latanoprost was associated with a modest reduction in OSDI and maintained short-term IOP control. Objective conjunctival redness increased substantially, but NIKBUT showed no significant longitudinal change, burning improved, and netarsudil was preferred by 17/27 participants with available month-2 data, although preference was unknown for three noncompleters. Visible hyperemia and the overall patient experience should therefore be considered complementary rather than interchangeable outcomes. Larger controlled studies with longer follow-up are needed to determine whether reduced medication and instillation burden produces sustained ocular-surface, adherence, or preference benefits.

## Figures and Tables

**Table 1:** Longitudinal objective ocular outcomes after switching to netarsudil.

Outcome	Baseline	Month 1	Month 2	Overall Visit *p* Value
**Serle hyperemia grade, *n* (%)**				
Grade 0	1 (1.9%)	0	0	—
Grade 1	49 (90.7%)	26 (48.1%)	30 (55.6%)	—
Grade 2	3 (5.6%)	26 (48.1%)	18 (33.3%)	—
Grade 3	1 (1.9%)	2 (3.7%)	6 (11.1%)	—
Grade ≥ 2	4 (7.4%)	28 (51.9%)	24 (44.4%)	<0.0001
BRI	1.57	1.99	2.14	<0.0001
TMH, mm	0.275	0.359	0.329	<0.0001
NIKBUT, s	7.17	8.76	7.34	0.2154
IOP, mmHg	14.5	14.33	13.65	0.0083
BCVA, logMAR	0.142	0.127	0.124	0.0215

Serle grades are presented as observed counts and percentages among 54 eyes at each visit. The overall effect for grade ≥ 2 hyperemia was evaluated using a marginal logistic model with participant-clustered standard errors. Continuous outcomes are presented as linear mixed-model estimated means. Models included 159 observations for BRI, 160 for TMH and NIKBUT, and 162 for IOP and BCVA; missing values were not imputed. Visit was modeled as a categorical fixed effect, with random intercepts for participants and eyes nested within participants. BRI, bulbar redness index; TMH, tear meniscus height; NIKBUT, non-invasive keratograph break-up time; IOP, intraocular pressure; BCVA, best-corrected visual acuity.

**Table 2:** Patient-reported comparison of ocular symptoms between netarsudil and the previous fixed dorzolamide/timolol drop at month 2 (*n* = 27).

Symptom	Previous Drop, Mean	Netarsudil, Mean	Improved with Netarsudil	No Change	Worsened with Netarsudil	Unadjusted *p* Value	Holm-Adjusted *p* Value
Itching	1.33	0.8	8 (29.6%)	17 (63.0%)	2 (7.4%)	0.018	0.073
Foreign-body sensation	0.72	0.59	9 (33.3%)	13 (48.1%)	5 (18.5%)	0.549	1
Burning	1.39	0.39	12 (44.4%)	14 (51.9%)	1 (3.7%)	0.0049	0.024
Blurred vision	0.57	0.65	4 (14.8%)	17 (63.0%)	6 (22.2%)	0.677	1
Red eye	0.46	0.89	2 (7.4%)	15 (55.6%)	10 (37.0%)	0.096	0.287

Symptom intensity was rated from 0 to 5, with higher scores indicating greater severity. Bilateral numerical responses were averaged within each participant; when only one eye-specific response was available, that value was retained. Improvement and worsening were defined according to the direction of the participant-level score difference. Paired scores were compared using Wilcoxon signed-rank tests, with Holm adjustment across the five symptom comparisons.

## Data Availability

The de-identified data supporting the findings of this study are available from the corresponding author upon reasonable request, subject to ethics and institutional restrictions.

## Abbreviations

The following abbreviations are used in this manuscript:

CIs	confidence intervals
BRI	bulbar redness index
IOP	intraocular pressure
logMAR	logarithm of the minimum angle of resolution
NIKBUT	non-invasive Keratograph tear-film break-up time
OSD	Ocular Surface Disease
OSDI	Ocular Surface Disease Index
TMH	tear meniscus height
